# pSTR Finder: a rapid method to discover polymorphic short tandem repeat markers from whole-genome sequences

**DOI:** 10.1186/s13323-015-0027-x

**Published:** 2015-08-05

**Authors:** James Chun-I Lee, Bill Tseng, Bing-Ching Ho, Adrian Linacre

**Affiliations:** Department of Forensic Medicine, College of Medicine, National Taiwan University, No. 1 Jen-Ai Road Section 1, Taipei, 10051 Taiwan; Department of Clinical Laboratory Sciences and Medical Biotechnology, College of Medicine, National Taiwan University, No. 1 Jen-Ai Road Section 1, Taipei, 10051 Taiwan; NTU Center for Genomic Medicine, College of Medicine, National Taiwan University, No. 1 Jen-Ai Road Section 1, Taipei, 10051 Taiwan; School of Biological Sciences, Flinders University, Adelaide, 5001 Australia

**Keywords:** STR, Bioinformatics, Whole-genome sequences, MASSIVE parallel sequencing, Forensic, TRF, FASTA

## Abstract

**Background:**

Whole-genome sequencing is performed routinely as a means to identify polymorphic genetic loci such as short tandem repeat loci. We have developed a simple tool, called pSTR Finder, which is freely available as a means of identifying putative polymorphic short tandem repeat (STR) loci from data generated from genome-wide sequences. The program performs cross comparisons on the STR sequences generated using the Tandem Repeats Finder based on multiple-genome samples in a FASTA format. These comparisons generate reports listing identical, polymorphic, and different STR loci when comparing two samples.

**Methods:**

The web site http://forensic.mc.ntu.edu.tw:9000/PSTRWeb/Default has been developed as a means to identify polymorphic STR loci within complex mass genome sequences. The program was developed to generate a series of user-friendly reports.

**Results:**

As proof of concept for the program, four FASTA genome sequence samples of human chromosome X (AC_000155.1, CM000685.1, NC_018934.2, and CM000274.1) were obtained from GenBank and were analyzed for the presence of putative STR regions. The sequences within AC-000155.1 were used as an initial reference sequence from which there were 5443 identical and 4305 polymorphic STR loci identified using a repeat unit of 1–6 and 10 bp as the flanking sequence either side of the putative STR loci. A reliability test was used to compare five FASTA samples, which had sections of DNA sequence removed to mimic partial or fragmented DNA sequences, to determine whether pSTR Finder can efficiently and consistently find identical, polymorphic, and different STR loci.

**Conclusions:**

From the mass of DNA sequence data, the project was found to reproducibly identify polymorphic STR loci and generate user-friendly reports detailing the number and location of these potential polymorphic loci. This freely available program was found to be a useful tool to find polymorphic STR within whole-genome sequence data in forensic genetic studies.

**Electronic supplementary material:**

The online version of this article (doi:10.1186/s13323-015-0027-x) contains supplementary material, which is available to authorized users.

## Background

Microsatellites, also known as short tandem repeat (STR) loci, are abundant in eukaryotic genomes [1]. Their polymorphic nature makes them suitable for use in population biology, especially in forensic science and parentage testing [2]. The search for polymorphic STR loci can be laborious and time-consuming using conventional means for any species for which little genome sequence is known [3]. The advent of massive parallel sequencing technology has led to the development of a number of search tools for identifying STR loci [4–7]. Despite these recent developments, it is a far from simple task to identify polymorphic STR loci from a mass of DNA sequence data [8, 9]. This process is still labor intensive, and few software programs are available to assist with identifying whether putative STR loci are polymorphic and suitable for further inclusion in any multiplex. Additionally, it is often very difficult, if not impossible, to detect all potential STR sequences in the whole-genome sequence data.

There are a number of software program developed for this purpose particularly when dealing with data from massive parallel sequencing such as MyFLq [10], lobSTR [11], and RepeatFinder [12], while some programs are useful in identifying a putative STR, not all are designed to indicate whether the locus is polymorphic and able to pullout the flanking DNA.

We have developed pSTR Finder (pSTR) to efficiently analyze multiple-genome sequence samples for the presence of STR loci using Tandem Repeats Finder (TRF) [12]. pSTR accepts sample data in the FASTA format and utilizes TRF. The pSTR program is then designed to analyze all input samples to discover and record putative polymorphic STR loci, regardless of whether the input sample was complete, or fractions of, a genome. We have found this program to be highly efficient when screening for potential polymorphic STR loci from genome-wide sequences and a major improvement on the current situation such that polymorphic STR loci can be identified rapidly from a large dataset.

## Methods

pSTR is a web application available for non-commercial use (http://forensic.mc.ntu.edu.tw:9000/PSTRWeb/Default.aspx). pSTR requires STR data generated using TRF. The most recent release of TRF that has been tested and integrated with pSTR is version 4.07b.

pSTR relies on TRF to adequately generate user-desired sizes (1–10 bp) of repeat sequences as the input data. While the user interface for pSTR is intuitive, it is possible to download and run either the desktop or command line version of TRF using the desired specific options and then submit the results of the TRF-generated repeat sequences to pSTR for processing.

In regard to the requirements for the input of TRF data, the user submits multiple FASTA contig sequences from which one sequence file is designated as the sample reference. Currently, the program is designed with the characterization of STR loci in mind, and therefore, users can specify the size of the 5′ and 3′ flanking sequences. These flanking sequences are used by pSTR to compare two sample sequences entered.

At the end of the pSTR matching process, multiple reports will be generated and saved in the comma separated values (CSV) format.

1. A summary report shows the number of identical STR loci, the number of polymorphic STR loci, and the number of different STR loci between two matching samples. The summary report also shows the total number of identical (or matching in repeat motif) STR loci, polymorphic STR loci, and unique (identified only once) STR loci for all samples analyzed, based on comparison between the tested sequence file and the designated reference sample file.
2. The detailed report includes in an CSV format: the 5′ and 3′ flanking sequences, the number of bases constituting the repeat unit, the sequence of the STR motif, the position of the first base in the STR repeat, the variation in the number of repeat units based on the samples included and with the same 5′ and 3′ flanking sequences, the total number of STR loci within the sample, and the number of repeats for every other sample included. Currently, excluded in this detailed report are STR sequence records that are duplicates such as where there is the same putative STR locus recorded but having within it a possible shorter repeat motif and STR sequence records having the same 5′ and 3′ flanking sequences but a smaller number of repeats. These two types of STR sequence, excluded from the detailed report, are saved for each sample in the duplicated STR report.
3. Duplicated STR report: a separate report for each sample captures the ‘duplicated’ STR records described above.
4. Identical STR report: this report captures all ‘identical’ STR sequence records from the comparison of two samples. An ‘identical’ STR is determined as having both the same flanking sequences and the repeat number for any two matching STR loci within the two input samples.
5. Polymorphic STR report: this report captures all STR sequence records having the same flanking sequences but a different repeat number between two input samples.
6. Different STR report: this report, like 4 and 5 above, requires only two input samples being the source sample of interest and any target file. This report captures all STR sequence records that exist in the source sample only and not in the other sequence file.
7. Different STR report after switching samples: this report also captures all ‘different’ STR sequence records when the ‘source’ and ‘target’ samples are switched and then re-matched.

The performance of pSTR was tested using four sets of sequences of human chromosome X (AC_000155.1, CM000685.1, NC_018934.2, and CM000274.1) from GenBank. There are 143,733,266, 155,270,560, 155,181,468, and 155,407,050 bp in the respective FASTA source samples.

The TRF options selected/entered to search all four chromosome X samples are below:

- Alignment Score for match, mismatch, indel: 2, 7, 7
- Minimum Alignment Score: 50
- Maximum Repeat Unit Size: 6
- 5′ and 3′ Flanking Sequence Size: 10

A reliability test was performed using AC_000155.1 as the reference sample. To mimic fragmented or partial DNA sequences, AC_000155.1 was divided into between four DNA fragments each comprising 5–10 Mbp. These new files were called AC_000155.1a, AC_000155.1b, AC_000155.1c, and AC_000155.1d.

## Results and discussion

As a result of searching the FASTA contig sequences of four X-chromosome samples with access number AC_000155.1, CM000685.1, NC_018934.2, and CM000274.1, the TRF found 14,026, 16,936, 15,891 and 16,698 potential STR loci, respectively. Loci that were found to be duplicates, i.e., those with the same repeat motif and the same flanking sequences, were saved to the specific duplicated STR report prior to matching. It should also be noted that the number and existence of false counts of identical STR loci, polymorphic STR loci, and different STR loci depends on the size of 5′ and 3′ flanking sequences specified (in this case, only ten were used).

A total number of 30 reports were generated by pSTR that included: 1 summary report, 1 detail report, 4 duplicated STR reports, and 24 matching reports. The summary report generated in this example is shown in Table 1. One example of each of the reports is available as Additional file 1. Using AC_000155.1 as the reference data and comparing the other data, there were 5443 identical STR loci, 4305 polymorphic STR loci, and 0 unique STR loci among samples. There were in total 12,518 STR loci captured in the detailed report.

**Table 1:** Summary results of the number of identical, polymorphic and different STR loci among four samples after searching using pSTR

Samples	AC_000155.1	CM000685.1	NC_018934.2	CM000274.1
AC_000155.1		7034 (4654)	6716 (4096)	8592 (2906)
CM000685.1	4935 (2197)		10720 (2790)	10930 (3017)
NC_018934.2	4807 (3073)	3113 (2109)		8655 (3546)
CM000274.1	5033 (2387)	2676 (2584)	4330 (3418)	

Figures in the upper quadrant indicate the number of identical STR loci with the total number of polymorphic STR loci in brackets. The figures in the lower quadrant indicate the number of different STR loci, and the numbers in brackets indicate the number of different STR loci after switching the ‘source sample’ with the ‘target sample’. Using AC_000155.1 as the reference data and comparing the other data, there are 5443 identical STR loci, 4305 polymorphic STR loci and 0 unique STR loci (please see Additional file 1 for further information)

The reliability test resulted in fewer polymorphic STR being observed as all samples are a ‘subset’ of the reference sample AC_000155.1 (see Table 2). In addition, if the length of the 5′ and 3′ flanking size was increased from 10 to 100 bp, then the polymorphic STR count of each comparison of two samples becomes 0 (see Table 3).

**Table 2:** Summary results of the number of identical, polymorphic and different STR loci among five samples after searching using pSTR with 10 bp flanking sequences used as a reliability test

Samples	AC_000155.1	AC_000155.1a	AC_000155.1b	AC_000155.1c	AC_000155.1d
AC_000155.1		10856 (3)	11137 (9)	10440 (4)	10793 (12)
AC_000155.1a	3026 (0)		8108 (12)	9395 (4)	8734 (13)
AC_000155.1b	2739 (0)	3026 (2739)		7692 (13)	8045 (21)
AC_000155.1c	3441 (0)	1460 (1045)	3441 (2739)		7348 (16)
AC_000155.1d	3080 (0)	2112 (2058)	3080 (2739)	3441 (3080)	

Figures in the upper quadrant indicate the number of identical STR loci with the total number of polymorphic STR loci in brackets. The figures in the lower quadrant indicate the number of different STR loci and the numbers in brackets indicate the number of different STR loci after switching the ‘source sample’ with the ‘target sample’

**Table 3:** Summary results of the number of identical, polymorphic and different STR loci among five samples after searching using pSTR with 100 bp flanking sequences used as a reliability test

Samples	AC_000155.1	AC_000155.1a	AC_000155.1b	AC_000155.1c	AC_000155.1d
AC_000155.1		10927 (0)	11211 (0)	10502 (0)	10861 (0)
AC_000155.1a	3047 (0)		8164 (0)	9454 (0)	8785 (0)
AC_000155.1b	2763 (1)	3048 (2763)		7739 (0)	8098 (0)
AC_000155.1c	3472 (0)	1473 (1048)	3473 (2763)		7389 (0)
AC_000155.1d	3113 (0)	2142 (2076)	3114 (2763)	3472 (3113)	

Figures in the upper quadrant indicate the number of identical STR loci with the total number of polymorphic STR loci in brackets. The figures in the lower quadrant indicate the number of different STR loci and the numbers in brackets indicate the number of different STR loci after switching the ‘source sample’ with the ‘target sample’

The summary report (see Table 1) provides a quick view of the number of identical and polymorphic STR loci, how many potentially different STR were found, and how many STR loci were found to be unique. A record of these findings are grouped and saved in the detailed report. Additionally, the following is provided: different STR report, identical STR report, and polymorphic STR report. If required, users may utilize Excel pivot tables and graphs or other tools as an advanced method to analyze grouped STR loci in any of the reports generated by the pSTR program. For instance, the flanking sequences saved in the reports can be used as the sources of PCR primers for the development of a STR genotyping system.

There are between 2790 and 4654 polymorphic STR based on the comparison of any two samples found by the pSTR program. All STR loci identified as exhibiting variation in the number of repeats based on the comparison of two samples are saved in the polymorphic STR report. Likewise, all identical STRs after the comparison of two samples are saved in the identical STR report.

The duplicated STR report captures all the STR records that are at the same position but have different annotated STR sequences and also the STR records having the same 5′ and 3′ flanking sequences but found at different positions. For instance, in the duplicated STR report of sample AC_000155.1, there are 54 clusters containing two STR loci recorded as being at the same position. Further, there are 157 clusters containing between 2 and 14 STRs having the same flanking sequences. In addition, there are 35 clusters having the same position and also the same flanking sequences. pSTR effectively minimizes the false data commonly seen in genome-wide sequences due to errors created during the assembling of the data by excluding all the duplicated STR from the duplicated STR reports.

The different STR report, generated by analyzing two samples, captures all the STR loci that exist in one sample, called the ‘source sample’, but do not exist in another sample called the ‘target sample’. For the same pair of matching samples, a separate different STR report can be generated if the source sample and target sample are switched. The performance test found between 2109 and 5033 different STR loci as matches when two samples were compared. As this is a different STR report, then a smaller number suggests a higher similarity between the samples being compared.

No unique STR loci were found among four samples using ‘AC_000155.1’ as the reference sample in our performance test. A unique STR may be recorded if there is variation in the flanking sequences or variation in a repeat sequence. It is also possible that the presence of a unique STR locus was due to sequencing or assembling errors.

In the reliability test, since all samples are a ‘subset’ of the reference sample AC_000155.1, no polymorphic STR loci should be observed in the summary report. In Table 2, however, there are a few polymorphic STR loci recorded. Analysis of the data found that these are all artifacts of the sequences over the conjunctions of DNA contigs in AC_000155.1a to d. For the same reason, no ‘different STR’ was found while comparing all the ‘subset’ samples with the reference sample, an exception being between AC_000155.1b, and AC_000155.1 where 1 is recorded in Table 3.

As pSTR uses the 5′ and 3′ flanking sequences to compare between two input samples, the accuracy of identification of polymorphic STR loci can be increased by increasing the length of the flanking DNA. For instance, by increasing the 5′ and 3′ flanking size from 10 to 100 bp, the polymorphic STR count of each matching of two samples became 0.

Examples of all these reports can be found in the Additional file 1.

## Conclusions

The program pSTR was found to efficiently analyze multiple TRF generated data to accurately identify putative STR loci. The program will generate reports detailing different, identical, and polymorphic STR data from all the input samples. The concept was to design a useful and valuable tool that is easy to use when searching for polymorphic and unique STRs from genome-wide sequences.

Recently published programs such as RepeatFinder [12, 13], lobSTR [11], and myFLq [10] are very effective at identifying putative STR loci and are an advance of prior methods when examining a plethora of data generated by massively parallel sequencing. pSTR not only identifies putative STR loci but also generates numerous reports detailing the polymorphic nature of the loci such that these loci may be suitable for further genetic testing.

A practical use of pSTR relevant to forensic practice is to identify variations of STR loci between two whole-genome sequences from the same sample type but of different size or complexity. An example of which is a DNA sample from a known person, where a complete genome is available from a near pristine sample, and an evidential sample collected from a scene, where the size of genetic data may be limited or compromised.

Another practical use of pSTR is to search SNPs within whole-genome sequence data to differentiate identical twins [14]. Since the mutation rate of some STR loci is approximately 100,000 times higher than some SNPs [15], there would be a high expectation of identifying STRs that separate even identical twins. This program can work on sections of a genome or a complete genome to identify polymorphic STR loci that may distinguish such monozygotic twins. The program pSTR can also find SNPs by searching large flanking sequences from data obtained from such identical twins.

It is intended that this program will aid in the easy analysis of data generated from whole-genome sequencing and become a labor-saving program.

## Additional file

Additional file 1:
**STR matching summary report.** STR matching detail report. STR matching duplicate report. STR matching reference sample [sample 2]. Different STR (switched sample). STR matching reference sample [sample 2]. Different STR. STR matching reference sample [sample 2]. Identical STR. STR matching reference sample [sample 2]. Polymorphic STR. (ZIP 1015 kb)

## Abbreviations

CSV: comma separated values is a file format
FASTA: text-based format for DNA and protein sequences used in bioinformatics
pSTR: the abbreviation used for pSTR Finder, the software described in this paper
short tandem repeat: a form of hypervariable DNA sequence where the repeat motif occurs in a tandemly repeated manner and the number of repeats is variable between individuals
TRF: Tandem Repeats Finder is a software tool to identify potential repetitive polymorphic DNA sequences
